# The effects of DNA formulation and administration route on cancer therapeutic efficacy with xenogenic EGFR DNA vaccine in a lung cancer animal model

**DOI:** 10.1186/1479-0556-7-2

**Published:** 2009-01-30

**Authors:** Ming-Derg Lai, Meng-Chi Yen, Chiu-Mei Lin, Cheng-Fen Tu, Chun-Chin Wang, Pei-Shan Lin, Huei-Jiun Yang, Chi-Chen Lin

**Affiliations:** 1 Department of Biochemistry and Molecular Biology, College of Medicine, National Cheng Kung University, Taiwan; 2 Institute of Basic Medicine, College of Medicine, National Cheng Kung University, Taiwan; 3 Center for Gene Regulation and Signal Transduction Research, National Cheng Kung University, Tainan, Taiwan; 4 School of Medicine, College of Medicine, Taipei Medical University, Taipei, Taiwan; 5 Department of Emergency Medicine, Shin Kong Wu Ho-Su Memorial Hospital, Taipei, Taiwan, R.O.C; 6 Institute of Medical Technology, College of Life Science, National Chung Hsing University, Taiwan; 7 Department of Medical Research and Education, Taichung-Veterans General Hospital, Taichung, Taiwan

## Abstract

**Background:**

Tyrosine kinase inhibitor gefitinib is effective against lung cancer cells carrying mutant epidermal growth factor receptor (EGFR); however, it is not effective against lung cancer carrying normal EGFR. The breaking of immune tolerance against self epidermal growth factor receptor with active immunization may be a useful approach for the treatment of EGFR-positive lung tumors. Xenogeneic EGFR gene was demonstrated to induce antigen-specific immune response against EGFR-expressing tumor with intramuscular administration.

**Methods:**

In order to enhance the therapeutic effect of xenogeneic EGFR DNA vaccine, the efficacy of altering routes of administration and formulation of plasmid DNA was evaluated on the mouse lung tumor (LL2) naturally overexpressing endogenous EGFR in C57B6 mice. Three different combination forms were studied, including (1) intramuscular administration of non-coating DNA vaccine, (2) gene gun administration of DNA vaccine coated on gold particles, and (3) gene gun administration of non-coating DNA vaccine. LL2-tumor bearing C57B6 mice were immunized four times at weekly intervals with EGFR DNA vaccine.

**Results:**

The results indicated that gene gun administration of non-coating xenogenic EGFR DNA vaccine generated the strongest cytotoxicty T lymphocyte activity and best antitumor effects. CD8(+) T cells were essential for anti-tumor immunityas indicated by depletion of lymphocytes in vivo.

**Conclusion:**

Thus, our data demonstrate that administration of non-coating xenogenic EGFR DNA vaccine by gene gun may be the preferred method for treating EGFR-positive lung tumor in the future.

## Background

The epidermal growth factor receptor (EGFR) is a transmembrane glycoprotein, which consists of three domains: an extracellular ligand-binding domain that recognizes and binds to specific ligands, a hydrophobic membrane-spanning region, and an intracellular catalytic domain that serves as the site of tyrosine kinase activity [1,2]. High EGFR protein expression was observed in several types of cancer including breast, bladder, colon and lung carcinomas [3-6]. This involvement in cancer progression and a negative prognosis makes EGFR an attractive target for molecule therapy [7]. Various therapeutic strategies have been developed to block EGFR signaling, with the most frequent strategies involving monoclonal antibodies and small molecule tyrosine kinase inhibitors that are designed to directly against receptor or specifically inhibit EGFR enzymatic activity [8-10]. However, some clinical studies indicated that tumors overexpressing EGFR did not show a significant clinical response to antibody-based or small molecule inhibitor therapy in lung cancer, Searching for correlates, it has been found that the presence of certain kinase domain mutations in EGFR gene appear to predict responsiveness [11-13]. Hence, new strategies are required to treat tumors overexpressing normal EGFR.

Antigen-specific active immunotherapy is another potential therapeutic approach for the treatment of EGFR-positive tumor cells by breaking of immune tolerance against wild type or mutant-type EGFR. Since the anti-EGFR antibody was not effective, the active immunotherapy may need to induce both humoral and cellular immunity. DNA vaccine apparently fulfills such a requirement [14]. Furthermore, DNA vaccine offer many advantages including induction of a long-lived immune response, better stability, and easy preparation in large quantities than other conventional vaccines such as peptide or attenuated live or killed pathogens [15]. In addition, several studies have indicated that tolerance to self antigens on cancer cells can be overcome using active therapeutic immunization strategies in preclinical animal model [16,17].

Intramuscular administration of xenogenic EGFR DNA vaccine has been shown to break immune tolerance and induce the specific antitumor immunity against EGFR-positive tumors in a therapeutic preclinical model [18]. Two common routes of immunization have been for DNA vaccination: needle intramuscular injection and epidermal gene gun bombardment. Many studies have shown that gene gun-mediated immunization is more efficient than needle intramuscular injection as it elicits similar levels of humoral and cellular response [19,20]. However, intramuscular injection of DNA induces a predominantly Th1 response, whereas gene gun immunization with DNA coated on gold evokes mainly Th2 response. The route of immunization can influence the outcome of the immune response through altering the interaction between the vaccine and different APCs at the site of injection [21]. Our previous results suggested that gold particles used in gene gun bombardment affected the induced-immune response [22], because gene gun administration using non-coating naked DNA vaccine elicited Th1-bias immune response. Hence, the choice of the route of DNA immunizations and formulation of DNA could thus represent an important element in the design of EGFR DNA vaccine against EGFR-positive tumor.

In the present study, we aimed to determine how different route of administration and formulation of plasmid DNA could influence the efficacy of xenogenic EGFR DNA vaccine on a mouse lung tumor LL2 naturally overexpressing endogenous EGFR. We analyzed and compared the immunological and antitumor responses generated by the plasmid DNA encoding extracellular domain of human EGFR(a.a 1–621, Sec-N'-EGFR) administrated through three different methods: needle intramuscular administration using non-coating DNA (i.m), gene gun administration using gold-coated DNA and gene gun administration using non-coating DNA. Our results indicated that the routes of administration and formulation of DNA clearly affected the therapeutic response by altering immune pathway. Gene gun administration using non-coating plasmid DNA induced the best anti-tumor immune response in LLC2 animal lung cancer animal model, which may provide the basis for the design of DNA vaccine in human clinical trial in the future.

## Methods

### Animals, Cell lines and antibodies

Inbred female C57BL/6 mice (6–8 weeks of age) weighing 18–20 g were used. Animal experiments were approved by the National Cheng Kung University animal welfare committee. LL2 is a cell line derived from Lewis lung carcinoma passaged routinely in C57BL/6 mice [23]. B-16 F10 melanoma cell line and colon carcinoma cell line CT-26 were obtained from American Type Culture Collection (Manassas, VA, USA). Antibody against the extracellular domain of mouse EGFR (N20; Santa cruz) was used for Western blotting analysis of the expression of EGFR in these cell lines. Antibody against mouse extracellular EGFR (N20; Santa cruz) and FITC-conjugated donkey against goat IgG secondary Ab (Jackson Immuno Research Laboratories, Inc) were used for detection of surface EGFR in LL2 cells. Flow cytometry analysis was performed with a FACSCalibur (BD Bioscience, Mountain View, CA, USA).

### Construction and Preparation of DNA vaccine

A431 cells were harvested and total RNA was isolated using a total RNA extraction kit (Viogene-Biotek Corp., Hsichih, Taiwan) according to the manufacturer's instructions. The RNA was subjected to reverse transcriptase polymerase chain reaction (RT-PCR) for amplification of the extracellular domain of the human EGFR gene (Sec-N'-EGFR) using the primers GCAATCAAGCTTATGCGACCCTCCG GGACGG and GCAATCTCTAGACACA GGTGGCACACATGGCC The PCR product of the expected size was isolated, digested with HindIII and XbaI, and cloned into the multiple cloning site of pcDNA3.1B+myc-his (Invitrogen, San Diego, CA, USA). The plasmid DNA was transformed into Escherichia coli DH5 and purified from large-scale cultures using a QIAGEN Endofree Mega Kit (Qiagen, Chatsworth, CA, USA).

### In vitro transfection and Western blotting

COS-7 cells were transiently transfected with DNA plasmids by Lipofactamine 2000 (Invitrogen, San Diego, CA, USA), and cells were harvested 18 h post transfection. Total cell lysates were prepared by using 2× SDS gel loading buffer(Tris-HCl pH 8.45, 90 mM, Glycerol 24%, SDS 4%). Equal amounts of cell lysates (30 μg of total protein) were separated by sodium dodecyl sulfate-polyacrylamide gel electrophoresis and transferred onto PVDF membranes (minipore). The membrane was blocked for 1 h at room temperature in PBS containing 5% nonfat dried milk and 0.1% Tween 20 under gentle shaking. The membrane was then incubated overnight with EGFR-specific monoclonal antibody and the ound antibody was detected with a 1:2,000 dilution of horseradish peroxidase-conjugated goat anti-mouse immunoglobulin G (Cell Signaling Technology, Inc, Danvers, MA, USA). The immobilon Western chemiluminescent HRP substrate (Millipore Corporation, Billerica, U.S.A) was used for Western blotting. The intensity of each band was read by using a B UVP Biospectrum AC System (UVP, Upland, CA, U.S.A)

### Therapeutic efficacy of DNA vaccine on tumor growth

Mice were injected subcutaneously in the flank with 1 × 10^6 ^LL2 cells in 0.5 ml of PBS. At day 5, Sec-N'-EGFR DNA vaccine was administered by three different methods four times at weekly intervals when tumors were palpable. Control mice were injected with water containing no plasmid DNA. Tumor growth was monitored using caliper twice a week. Subcutaneous tumor volumes were calculated using the formula for a rational ellipse: (m1 × m2 × m2 × 0.5236), where m1 represents the longer axis and m2 the shorter axis. Mice were sacrificed when the tumor volume exceeded 2500 mm^3 ^or the mouse was in poor condition and death was expected shortly. Significance of differences in mice survival was tested by Kaplan-Meier analysis.

### DNA vaccination by needle intramuscular injection

For intramuscular needle-mediated DNA vaccination, 100 μg/mouse of Sec-N'-EGFR DNA vaccines or pcDNA3.1B+myc-his DNA plasmid were administered intramuscularly by syringe needle injection.

### DNA vaccination by gene gun gold-coated DNA or naked non-coating DNA

The protocol and delivery device for DNA vaccination by gene gun have been described previously [22]. Briefly, for gold-coated DNA vaccination, plasmid DNA was coated on gold particles (Bio-Rad, Hercules, CA, USA) at the ratio of 1–2 μg of DNA per mg of gold particles, and was dissolved in 20 μl of 100% ethanol. The gold-coated DNA was delivered to the shaved abdominal region of C57BL/6 mice using a helium-driven low pressure gene gun (Bio Ware Technologies Co. Ltd, Taipei, Taiwan) with a discharge pressure of 40 psi. For non-coating DNA vaccination, 1–2 μg of Sec-N'-EGFR DNA in 20 μl of autoclaved double-distilled water was directly added to the loading hole near the nozzle, and delivered to the shaved abdominal of mice using the same low pressure gene gun with a discharge pressure of 60 psi.

### Determination of anti-EGFR antibody titer in serum

Recombinant extracellular domain protein of human EGFR (0.25 μg/well) (R&D Systems Inc) in 100 μl coating buffer (sodium carbonate, pH 9.6) was added to microtiter plates (Nunc, Roskilde, Denmark) and incubated overnight at 4°C. Nonspecific binding was blocked with 1% BSA in PBS buffer for 2 h and washed with PBS containing 0.05% Tween 20 for three times. Mouse monoclonal anti-human EGFR antibody (20E12; Santa cruz) was used to generate the standard curve. The titer of anti-EGFR antibody in experimental mouse sera were determined by serial dilution and added to wells. Plates were incubated for 2 h at 37°C, washed, and then incubated with HRP-conjugated anti-mouse IgG (Cell Signaling Technology). TMB substrate was used for colour development. Absorbance was measured at 450 nm with an ELISA reader (Sunrise, Tecan, Austria).

### Serum passive transfer

LL2 tumor bearing B6 mice were immunized with DNA vaccine four times. Blood was collected 4, 7, 10 days after the last immunization, and serum was collected and pooled within each group of mice. A 300 μl of the polled sera was transferred by intraperitoneal injection into recipient mice which was s.c challenged with 1 × 10^6 ^LL2 tumor cells 5 day before. Blood collected from LL2 tumor bearing B6 mice without DNA vaccination was used as control.

### Intracellular staining

Spleen or lymph node cells(2.5 × 10^6 ^cells/ml) were harvested a day after last immunization and cultured in 48 well tissue culture plates (BD Biosciences) in the presence of 5 μg/ml of recombinant EGFR protein and incubated at 37°C in a 5% CO_2 _humidified atmosphere for 18 h. Thereafter, 5 μg/ml brefeldin A (BFA; Sigma, St. Louis, MO) was added, and the cultures were incubated for an additional 6 hr. Cells were harvested and stained with PE-anti-CD4 (eBioscience) and PE-anti-CD8 (eBioscience) and then fixed with 4% paraformaldehyde for 30 min at 4°C. The cells were permeabilized with PBS containing 0.1% saponin for 5 min, after which FITC-anti-IFN-γ (eBioscience) antibody was added for detection of intracellular cytokine in the presence of saponin for 45 min at 4°C. For analysis, 100000 cells were acquired on a Facscalibur. The results were analyzed using CellQuest (BD Biosciences).

### In vivo CTL assay

Spleen and inguinal lymph node cells from naive C57BL/6 mice were labeled with 5 or 0.5 μM CFSE. Cells labeled with 5 μM CFSE were pulsed with 5 μg/ml recombinant EGFR protein at 37°C for 1 hr as target cells while the cells labeled with 0.5 μM CFSE were left unpulsed as control cells. Equal number (1 × 10^7^) of the two target populations were mixed together and injected into mice i.v., such that each mouse was injected i.v with a total of 2 × 10^7 ^cells in 150 μl of PBS. Spleens and inguinal lymph nodes in recipient mice were harvested 18 hrs later and single-cell suspensions were prepared. The proportions of differentially CFSE-labeled target cells were analyzed by flow cytometry. To calculate specific lysis, the following formula was used: ratio = (percentage CFSE low/percentage CFSE high). Percentage of specific lysis = [1 - (ratio for unimmunized mice/ratio for immunized mice) × 100]

### Histological analysis of lymphocyte infiltration

Tumor tissues were removed from mice one week after the last vaccination and embedded in OCT compound (Sakura Finetek Inc., USA) and then frozen in liquid nitrogen. Cryosections (5-μm) were made and fixed with 3.7% formaldehyde and acetone. Endogenous peroxidase was removed with 3.7% hydrogen peroxide, washed with PBS three times and incubated with primary antibody anti-CD4 (GK1.5;BD Biosciences Pharmingen, San Jose, CA), or anti-CD8 (53-6.7; Pharmingen), overnight at 4°C. After further reaction with peroxidase-conjugated secondary antibody, aminoethyl carbazole substrate kit (Zymed Laboratories, San Francisco, CA) was used for color developing. For quantification of immune infiltrating cells, the cells were counted with a light microscope with a 10× eyepiece and a 40× objective lens. Three samples from three mice were taken and analyzed for statistical significance test.

### Depletion of CD8+ or CD4+ T cells

T cell-depletion experiments have been described previously[16]. Briefly, C57BL/6 mice were injected i.p with rat anti-mouse CD8 (2.43; 500 g), rat anti-mouse CD4 (GK 1.5; 300 μg), or control antibody (purified rat IgG; 500 μg). The depletions started 2 day before DNA vaccination, followed by multiple injections at 7-day intervals. To confirm the efficiency of T cell depletion, flow cytometry analysis revealed that the >95% of the appropriate subset was depleted

### Statistical Analysis

The animal experiments to evaluate immune responses were repeated at least two times (*n *= 3 per group). SE values were calculated with GraphPad Prism 4 software (GraphPad Software; San Diego, CA, USA), and P value less than 0.05 was considered statistically significant. Comparison of the survival rate was carried out by using Kaplan-Meier method and log-rank test in GraphPad Prism 4 software.

## Results

### The expression of EGFR in Mouse Cancer Cell Lines

**Figure 1:**
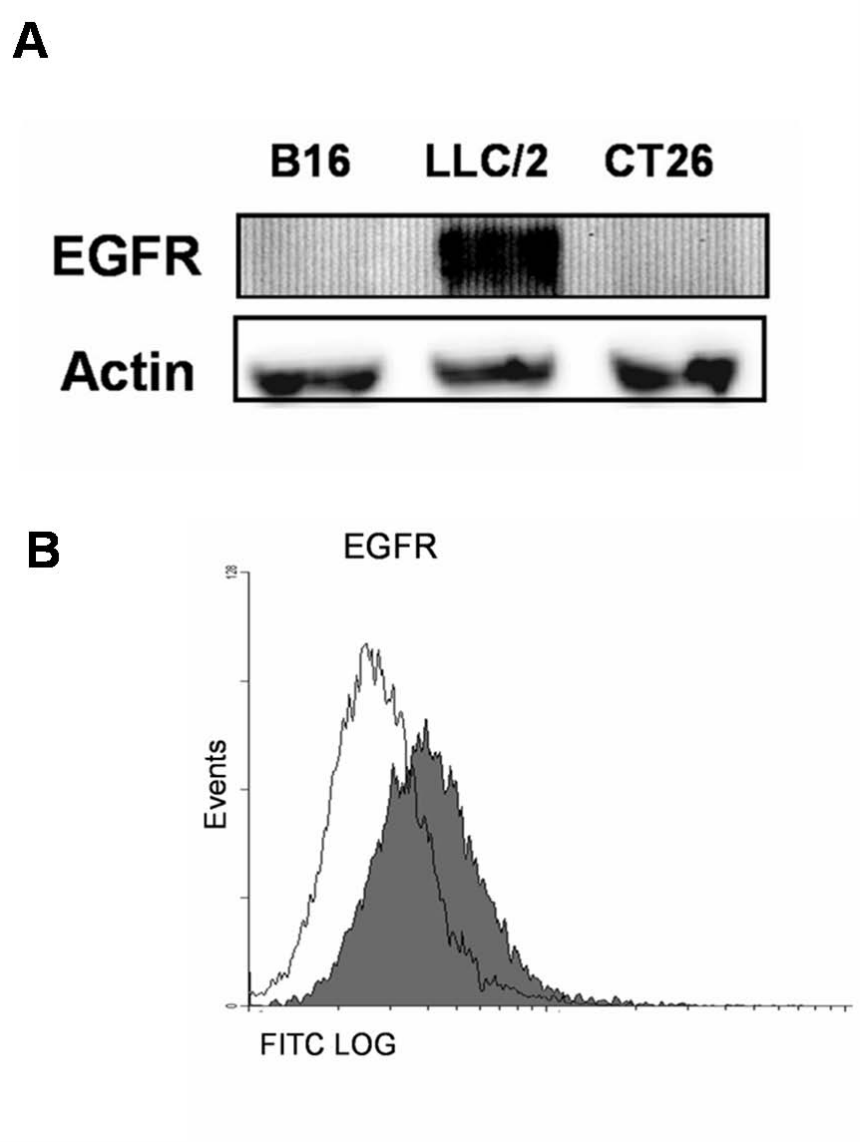
**Overexpression of EGFR in LL2 lung cancer cell line**. The expression of EGFR in various cell lines was analyzed by Western blotting with monoclonal antibody against EGFR. (B) Flow cytometry analysis of membrane EGFR in LL2 cells. LL2 cells were stained with monoclonal antibody against the extracellular domain of mouse EGFR, followed by FITC-conjugated mouse anti-goat secondary antibody (gray histogram). Normal mouse IgG mAb was used as the negative control (white histogram).

The expression of EGFR in several cancer cell lines was determined by Western blotting using antibodies that recognize the N-terminus of mouse EGFR (Fig. 1A). The expression of EGFR in LL2 lung tumor cells was the highest among three cell lines examined. In addition, we further confirmed surface expression of EGFR with flow cytometry (Fig. 1B). Therefore, the LL2 lung tumor in B6 mice is a good animal model to study the efficacy of the EGFR DNA vaccine.

### Construction and Characterization of Sec-N'-EGFR DNA vaccine

**Figure 2:**
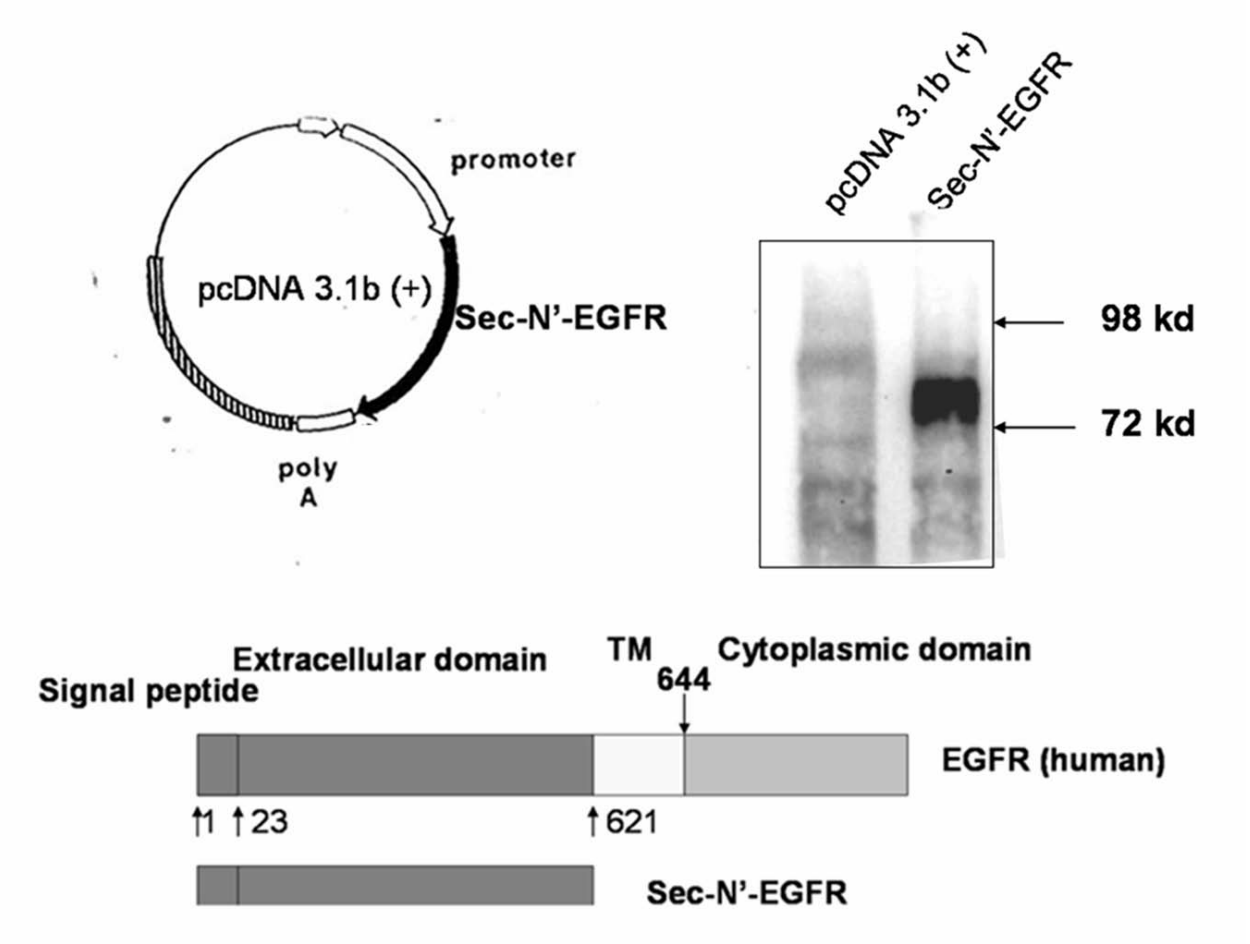
**Characterization of Sec-N'-EGFR DNA vaccines**. (A) Schematic diagram of the Sec-N'-EGFR expressing vectors. The N-terminal extracellular portion of the human EGFR gene was constructed to pcDNA3.1B+myc-his plasmid. Transcription is directed by cytomegalovirus (CMV) early promoter/enhancer sequences. The plasmid was named Sec-N'-EGFR (B) Expression of Sec-N'-EGFR was evaluated with transient transfection into COS-7 cells in vitro., and western blot analysis of sec-N-terminal EGFR protein. Whole cell lysates were collected from Cells transfected with Sec-N'-EGFR (lane 2), or control pcDNA3.1B+myc-his plasmid (lane 1), and analyzed with western blotting.

We first constructed the plasmid encoding the N-terminal extracellular domain of human EGFR (a.a. 1–621) and named the plasmid "Sec-N'-EGFR" (Fig. 2). The COS-7 cells were transfected with Sec-N'-EGFR DNA and the expression of extracellular domain of human EGFR was determined with western blotting. The Sec-N'-EGFR DNA plasmids expressed the extracellular domain of human EGFR in vitro (Fig. 2).

### Efficacy of Sec-N'-EGFR DNA Vaccine in Mice with Established Tumors

**Figure 3:**
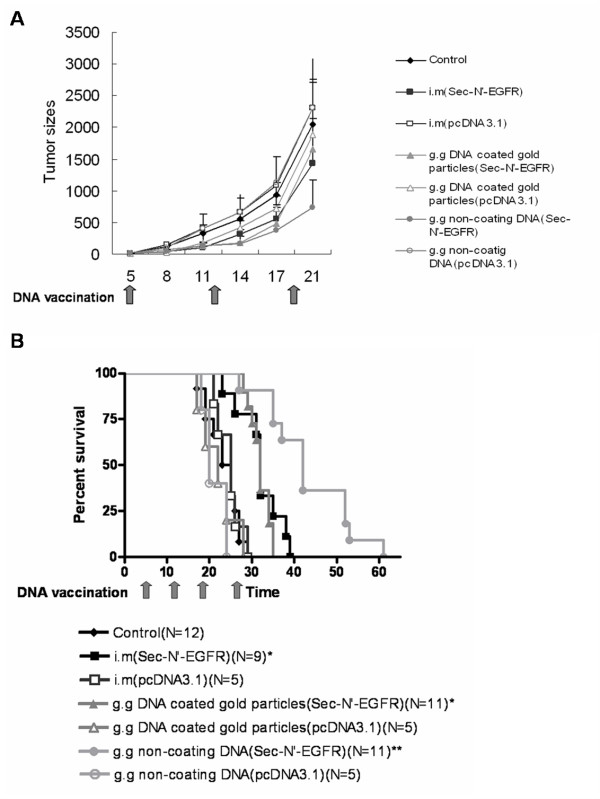
**Therapeutic effects of Sec-N'-EGFR DNA vaccine administered by three different methods on established tumor in B6 mice**. Five days after subcutaneous tumor implantation with 1 × 10^6 ^LL2 tumor cells., mice were administrated with DNA vaccine four times (day 5, 12, 19, 26) at weekly intervals; (A) tumor volume was measured at the indicated time. Data are means of the animals per group; bars, ± S.D. (B) lifespan of mice after subcutaneous challenge. The survival data were subjected to Kaplan-Meier analysis. The digit in the parenthesis is the number of mice in the experiment. The symbol (*) indicates a statistically significant difference when compared with the control saline mice (P < 0.01). The symbol (**) indicates a statistically significant difference when compared with the i.m and g.g gold-coated DNA group mice (P < 0.05) or control mice (P < 0.001). The experiments were repeated 2 times with similar results.

At day 0, we injected mice subcutaneously with 1 × 10^6 ^LL2 tumor cells. At day 5, when the tumor was palpable, we immunized the mice with Sec-N'-EGFR DNA vaccine four times at weekly intervals via three different methods: intramuscular injection (i.m), gene gun administration of gold-coated DNA, and gene gun administration of non-coating DNA. Non-coating Sec-N'-EGFR DNA vaccine administered by gene gun statistically delayed the growth of LL2 tumors when compared with control mice (Fig. 3A). In addition, the survival portion of vaccinated mice indicated that the therapeutic efficacy appeared to be in the order: g.g non-coating DNA vaccine mice group > g.g-DNA coated gold particels or i.m DNA vaccine mice groups >> control mice group (Fig 3B). The survival rate of mice showed significant differences between the control mice and all three vaccinated mice groups (p < 0.01). Furthermore, the difference between g.g non-coating DNA and the other two mice groups (i.m or g.g-DNA coated gold particles) is also statistically significant (P < 0.05)(Fig 3B)

### Humoral Immunity

**Figure 4:**
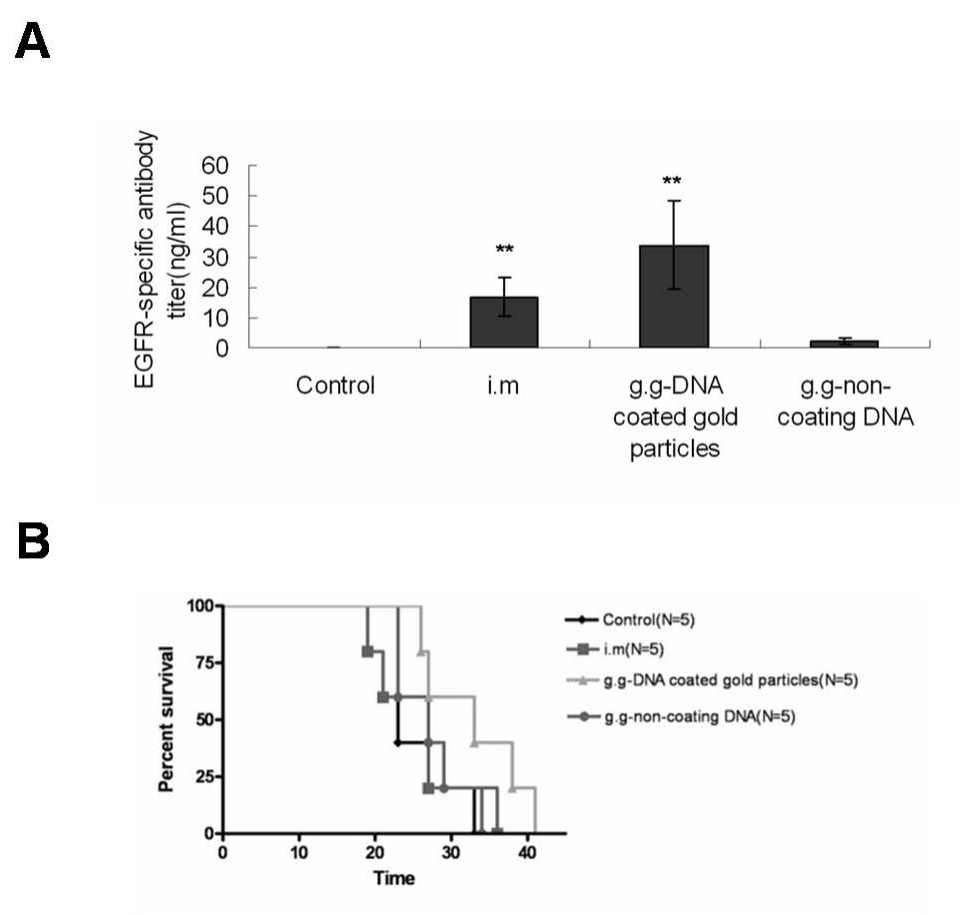
**The presence and the therapeutic efficacy of anti-EGFR antibody in serum from the DNA vaccine group of mice**. A) Anti-EGFR antibody titer in the mice serum. The serum anti-EGFR antibody in mice was determined with ELISA on dishes coated with the recombinant extracellular domain of human EGFR protein. The data represent the average titer of the sera from three mice in each group. The symbol (**) indicates a statistically significant difference when compared with the g.g non-coating DNA group mice (P < 0.05) or control mice (P < 0.001). B) B6 mice were treated with serum from control or vaccinated mice on day 5, 12, 19, 26 after s.c challenge with LL2 cells. The survival data were subjected to Kaplan-Meier analysis The symbol (*) indicates a statistically significant difference when compared with control mice group(P < 0.05). The experiments were repeated 2 times with similar results.

To investigate the immunological mechanism underlying the therapeutic effect of Sec-N'-EGFR DNA vaccine, the induction of anti-EGFR antibodies was examined in mice serum. Specific antibodies against EGFR proteins in mice serum samples were tested by ELISA using recombinant extracellular domain human EGFR proteins. The results showed that anti-EGFR antibodies were detected in all mice vaccinated with Sec-N'-EGFR DNA vaccine; however, the serum from g.g DNA coated gold particles and i.m mice groups contained higher levels of anti-EGFR antibodies than g.g-non-coating DNA mice group (Fig 4A). To further confirm the role of antibody in this therapeutic Sec-N'-EGFR DNA vaccine approach, the immune sera from mice vaccinated with DNA vaccine was passively transferred into mice with established LL2 tumors. The result showed that mice receiving serum from i.m mice group (p = 0.08) and g.g-DNA coated gold particles mice group(p < 0.05) showed prolong mice survival compared with mice injected with serum from control animals (Fig. 4B). The anti-EGFR antibody induced by Sec-N'-EGFR DNA played a role in delay tumor progression although the amount of antibody may not be correlated with antitumor effects of three forms of therapeutic EGFR DNA vaccine.

### Cellular Immunity

**Figure 5:**
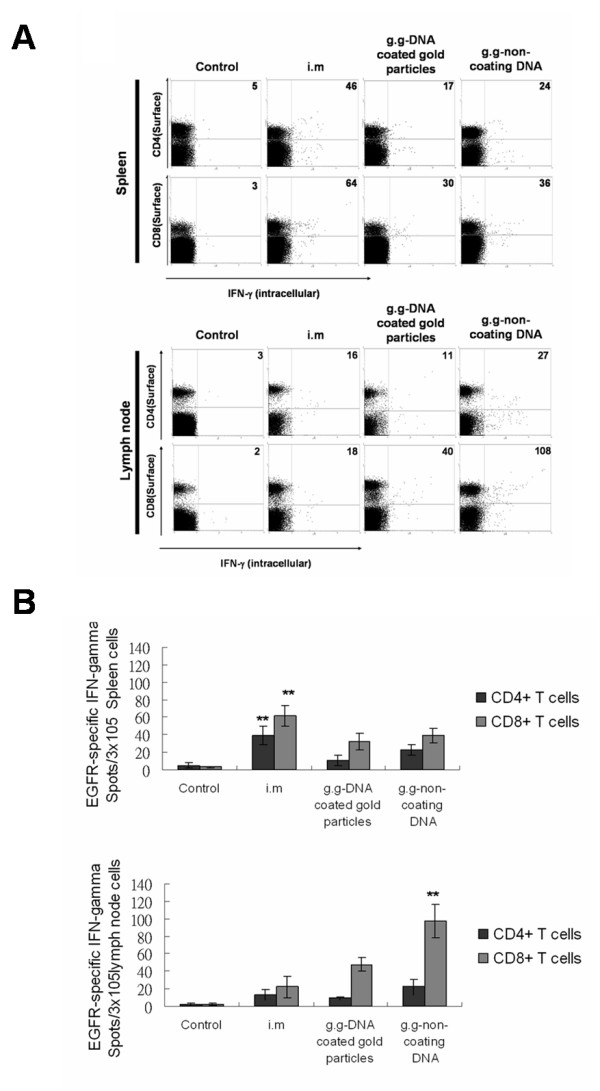
**Flow cytometry analysis EGFR-specific CD4^+ ^and CD8^+ ^T cells that functionally secrete IFN-γ in vaccinated mice**. A) the number of IFN-γ-producing EGFR-specific CD4+ and CD8+ T cells in both spleens and inguinal lymph node was determined using flow cytometry in the presence of recombinant extracellular domain of human EGFR. B). Data are expressed as the mean numbers of CD4^+ ^(black sqaure) and CD8^+ ^(black sqaure)IFN-γ^+ ^cells/3 × 105 spleen cells or inguinal lymph node cells; *bars*, SE. The symbol(**)indicates a statistically significant difference when compared with other treatment groups(P < 0.05). The data presented in this figure are from one representative experiment of two performed.

**Figure 6:**
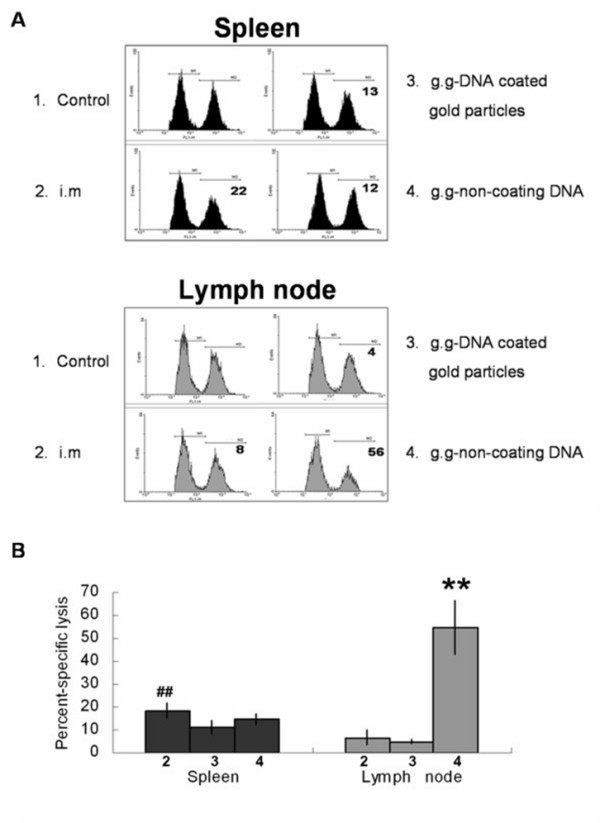
**In vivo CTL activity in vaccinated mice**. (A) In vivo EGFR-specific effector CTL are located throughout the secondary lymphoid system. A week after last DNA vaccination, an in vivo CTL using recombinant human EGFR protein pulsed splenocytes or inguinal lymph node as targets was performed to assess in vivo CTL activity. (B) The percentages of specific lysis were calculated to obtain a numerical value of cytotoxicity with data from each experimental group of three mice averaged. The symbol(##) and the symbol(**)indicates a statistically significant difference when compared with other treatment groups(P < 0.05). Similar results were obtained from two more repeated experiments (n = 3 per group).

To examine the specific immunologic cellular response to Sec-N'-EGFR DNA vaccine using different administration methods, spleen and lymph nodes were isolated from vaccinated mice. The lymphocytes were stained for the surface CD4 and CD8 marker and intracellular IFN-γ after recombinant human EGFR antigen stimulation. Non-coating Sec-N'-EGFR administration by gene gun generated most functional EGFR-specific CD8+ T cell cells as evidenced by their production of intracellular IFN-γ in the lymph node(Fig 5A, B). In contrast, splenic lymphocytes isolated from intramuscular injection of Sec-N'-EGFR mice group had higher functional EGFR-specific CD4+ and CD8+ T cells when compared with i.m and g.g DNA coated gold particles vaccinated mice groups, respectively(Fig 5A, B). In addition, we also measured cytotoxic T lymphocytes(CTLs) activity in mice immunized with Sec-N'-EGFR DNA vaccine by three different methods. The cytotoxic T lymphocytes(CTLs) effector function in spleen appeared to be in the order i.m mice group > g.g-DNA coated gold particles and g.g-non coating DNA mice groups>> control group (illustrated in an individual mouse in Fig. 6A and as group means in Fig. 6B). In contrast, the percent of specific cytotoxic T lymphocytes lysis in inguinal lymph node of vaccinated mice indicated that only non-coating Sec-N'-EGFR DNA administrated via gene gun is sufficient to induce CTL effector function (Fig 6A, B). Hence, taken together, the number of functional CD4+, CD8+ T cell and level of CTL activity in spleen and inguinal lymph node were differentially affected by the routes of administration and formulation of DNA vaccine.

**Figure 7:**
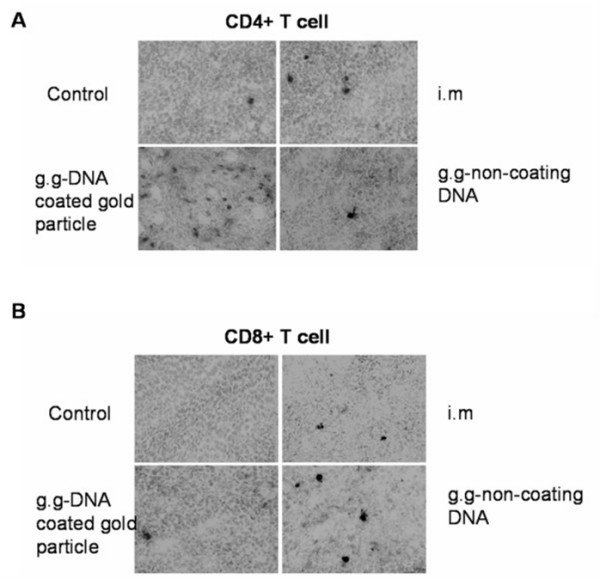
**Tumor infiltration of CD4+ and CD8+ T cells**. Tumors were excised from mice administrated with Sec-N'-EGFR, or control DNA vector by different delivery methods. Analysis of (A) CD4+ and (B) CD8+ T cells in cryosections of tumors was performed with staining with primary antibody specific for CD4+ and CD8+ cells respectively. Peroxidase-conjugated antibody was used as secondary antibody. Dark spots, peroxidase-stained cells. Similar results were obtained from two more repeated experiments (n = 3 per group).

To further demonstrate the importance of cellular immunity in cancer therapy, we examined the histology of the tumors. We observed CD4+ lymphocyte tumor infiltrations were detected in all mice groups (Fig. 7A and Table 1). However, tumors form g.g DNA coated gold particles mice group showed a greater infiltration of CD4+ lymphocytes compared with other treatment groups and control group. As for tumor infiltration of CD8+ T cell, we observed considerably increase of CD8+ lymphocyte in the g.g-non coating DNA mice group (Fig. 7B and Table 1) and minor increase of CD8+ lymphocytes in the i.m and g.g-gold mice group in comparison with control mice group. Hence, the results suggested a correlation between the therapeutic efficacy of gene gun administration of non-coating EGFR DNA vaccine and the amount of CD8+ T cell tumor infiltration.

**Table 1:** Infiltrated lymphocytes at tumor sites within cryosectioned samples

**Vaccine group**	**CD4^+ ^T cells**	**CD8^+ ^T cells**
Control	6 ± 1	0

i.m	17 ± 2**	11 ± 5^#^

g.g -DNA coated gold particles	118 ± 24***	4 ± 3^#^

g.g-non coating DNA	7 ± 2	23 ± 4^##^

Note: Cell count was performed at 400× magnification. Three samples and five randomly chosen fields/sample were evaluated. Results are expressed as mean ± standard deviation of immunohistochemical positive cells in the cryosection. GG: gene gun. The symbol (**) indicates a statistically significant difference when compared with the g.g non-coating DNA and control group (P < 0.05). The symbol (***) indicates a statistically significant difference when compared with the i.m and g.g non-coating DNA group (P < 0.05). The symbol (#) indicates a statistically significant difference when compared with the control group (P < 0.01). The symbol (##) indicates a statistically significant difference when compared with the i.m and g.g DNA coated gold particles group(P < 0.05).

### The effects of CD8+ T Cell- Depletion or CD4+ T Cell- De pletion on the Efficacy of Gene Gun Administration of Non-coating EGFR DNA vaccine

**Figure 8:**
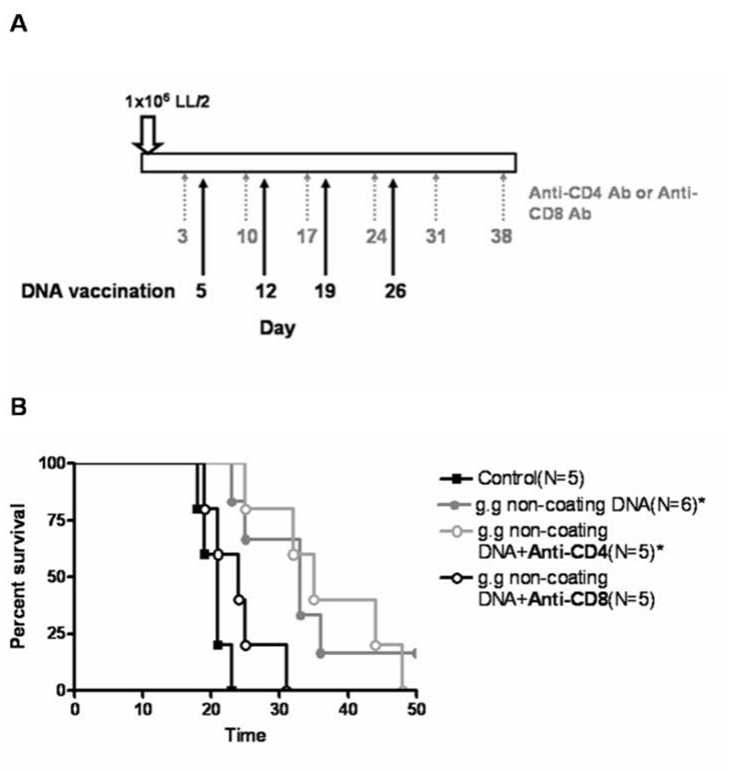
**The effects of CD8+ T cell-depletion or CD4+ T cell-depletion on the therapeutic effects of non-coating EGFR DNA vaccine by gene gun administration (A) Protocol for depletion of CD8+ or CD4+ T cells in vivo**. Tumor-bearing mice were injected i.p with 500 μg of anti-CD8 antibody or 300 μg of anti-CD4 antibody at weekly intervals starting from 2 days before the first inoculation of DNA vaccine. (B) Life span of B6 mice after sc challenge with LL2 tumor cells. ** represents statistically significant difference when compared to the control saline group of mice (P < 0.01). The experiments were repeated twice with experimental groups.

The efficacy of gene gun administration of non-coating EGFR DNA vaccine was the best among three types of EFEGFR DNA vaccines, and seemed to correlate with CD8+ T cells. Therefore, CD8+ T cells were depleted with monoclonal antibody 2.43 to determine whether CD8+ lymphocytes were required for the therapeutic efficacy. We performed CD8+ T cell-depletion at weekly intervals during the entire experiment, and the protocol is shown in Fig. 8A. Depletion of CD8+ lymphocytes completely abolished the therapeutic efficacy of Sec-N'-EGFR DNA vaccine delivered via g.g non-coating DNA method (Fig. 8B). On the other hand, it is known that CD4+ T cells have important regulatory functions for CD8+ CTL and antibody responses [24]. Hence, we also depleted CD4 +T cells with monoclonal antibodies GK1.5 and at weekly intervals during the entire experiment. The results showed that depletion of CD4+ T cell in mice did not affect the overall survival of mice administrated with non-coating DNA vaccine via g, g (Fig. 8B). Thus, these results suggested that CD8+ T cell played a major role in mediating therapeutic efficacy of gene gun administration of non-coating EGFR DNA vaccine

## Discussion

In this study, we assessed the immunologic responses and therapeutic antitumor effects of EGFR DNA vaccine delivered by three different methods: needle intramuscular administration using non-coating DNA (i.m), gene gun administration using DNA coated on gold particles (g.g-coated gold particles) and gene gun administration using non-coating DNA (g.g-non coating DNA) in an EGFR-overexpressing LL2 lung tumor animal model. Our results showed that non-coating Sec-N'-EGFR DNA vaccine administrated via gene gun represents the most potent regime for DNA administration. In addition, gene gun administration using non-coating Sec-N'-EGFR DNA vaccine generated higher EGFR-specific functional CD8+ T cell and EGFR-specific CTL activity in vivo comparing to other treatment groups. T cell-depletion experiment indicated that CD8+ T cell played a major role in mediating therapeutic efficacy of gene gun administration of non-coating EGFR DNA vaccine

In this study, we observed that gold-coated Sec-N'-EGFR DNA vaccine by gene gun generated higher antibody in the serum than g.g-non-coating DNA mice group. In addition, mice receiving serum from g.g-DNA coated gold particles mice group(p < 0.05) showed prolong mice survival when compared with mice injected with serum from control animals. These results suggested that anti-EGFR antibody produced also might be effective against EGFR-overexpressing tumor in LL2 model. However, clinical reports indicated that EGFR overexpression as detected by immunohistochemistry has not been correlated with response to small molecule EGFR inhibitor or anti-EGFR antibody therapy. The presence of certain EGFR kinase domain mutation appears to predict responsiveness better [11-13]. It is possible that the mutations present in EGFR precipitate the altered oncogenic signal and make EGFR indispensable for tumor growth, which make the inhibitor or antibody function to inhibit tumor growth. On the other hand, the reaction of CTLs does not depend whether the target molecules are indispensable or essential for the growth of tumor cells. Therefore, CTL effector cell may be useful against lung tumor with expressing wild type EGFR or mutant type EGFR protein. Hence, our results suggest that increase of Th1-like CTL immune response by administration of non-coating EGFR DNA vaccine may be the most potential application of EGFR DNA vaccine in the further clinical trials.

It was interesting to observe that administration route and forms of DNA affected the CTL activity in the spleen and inguinal lymph node differentially. Administration of Sec-N'-EGFR DNA vaccine by needle intramuscular injection can induce stronger CTL activity in spleen than gene gun administration of Sec-N'-EGFR DNA. In contrast, administration of non-coating Sec-N'-EGFR DNA vaccine via gene gun induced stronger CTL activity in inguinal lymph node than other treatments The different outcome of CTL activity may at least be explained by two reasons. First, i.m and g.g administrations of DNA may induce immune responses in different lymphoid compartments. Skin administration of DNA by gene gun appears to initiate responses by virtue of transfected epidermal Langerhans cells or antigen loaded epidermal Langerhans cells moving into draining inguinal lymph nodes [25-27]. By contrast, intramuscular injection of DNA initiated mainly by cells that has moved in the blood to the spleen [25,28]. Second, Th1 immunity is critical for the induction of specific cell-mediated cytotoxic cells including tumor-specific cytotoxic T lymphocytes in tumor-bearing mice. Our previous study demonstrated that administration of non-coating DNA via gene gun induced a predominantly T helper type 1 (Th1) response, whereas administration of gold-coated DNA by gene gun elicited predominantly T helper type 2 (Th2) responses [22]. Combination of these two factors may determine the final CTL activity in spleen and lymph nodes.

## Conclusion

In summary, the therapeutic efficacy of Sec-N'-EGFR DNA vaccine was dependent on the route of administration and formulation of plasmid DNA (gold-coating or non-coating). More importantly, we have shown that non-coating Sec-N'-EGFR DNA administration via gene gun represents the most potent regimen for EGFR DNA vaccine against EGFR-positive LL2 lung tumor and may be the preferred choice in the future clinical trial.

## Competing interests

The authors declare that they have no competing interests.

## Authors' contributions

MDL helped with the design of the experiments and prepared the draft manuscript, MCY, CFT, CCW, PSL and HJY performed the experiments; CML provided advice on the design of the study and commented on the manuscript; CCL conceived and supervised the study, participated in the preparation of and commented on the manuscript.
